# Focused Ultrasound-Induced Blood–Brain Barrier Opening Enhanced α-Synuclein Expression in Mice for Modeling Parkinson’s Disease

**DOI:** 10.3390/pharmaceutics14020444

**Published:** 2022-02-18

**Authors:** Chung-Yin Lin, Ching-Yun Huang, Chiung-Mei Chen, Hao-Li Liu

**Affiliations:** 1 Medical Imaging Research Center, Institute for Radiological Research, Chang Gung University, Taoyuan 33302, Taiwan; winwood7@mail.cgu.edu.tw (C.-Y.L.); sun01415@mail.cgu.edu.tw (C.-Y.H.); 2 Department of Nephrology and Clinical Position Center, Chang Gung Memorial Hospital, Taoyuan 33302, Taiwan; 3 Department of Neurology, Chang Gung Memorial Hospital, College of Medicine, Chang Gung University, Taoyuan 33302, Taiwan; 4 Department of Electrical Engineering, National Taiwan University, Taipei 10617, Taiwan; 5 Department of Biomedical Engineering, National Taiwan University, Taipei 10617, Taiwan

**Keywords:** ultrasound-targeted microbubble destruction, gene delivery, blood–brain barrier opening, α-synuclein, Parkinson’s disease

## Abstract

Parkinson’s disease (PD) is characterized by α-synuclein (αSNCA) aggregation in dopaminergic neurons. Gradual accumulation of αSNCA aggregates in substantia nigra (SN) diminishes the normal functioning of soluble αSNCA, leading to a loss of dopamine (DA) neurons. In this study, we developed focused ultrasound-targeted microbubble destruction (UTMD)-mediated PD model that could generate the disease phenotype via αSNCA CNS gene delivery. The formation of neuronal aggregates was analyzed with immunostaining. To evaluate the DA cell loss, we used tyrosine hydroxylase immunostaining and HPLC analysis on DA and its two metabolites, 3,4-dihydroxyphenylacetic acid (DOPAC) and homovanillic acid (HVA). This loss of DA was associated with a dose-dependent impairment in motor function, as assessed by the rotarod motor assessment. We demonstrate that UTMD-induced SNCA expression initiates αSNCA aggregation and results in a 50% loss of DA in SN. UTMD-related dose-dependent neuronal loss was identified, and it correlates with the degree of impairment of motor function. In comparison to chemical neurotoxin 1-Methyl-4-phenyl-1,2,3,6-tetrahydropyridine (MPTP)-treated and conventional intracerebral (IC)-injected animal models of PD, the UTMD-mediated αSNCA-based mouse model offers the advantage of mimicking the rapid development of the PD phenotype. The PD models that we created using UTMD also prove valuable in assessing specific aspects of PD pathogenesis and can serve as a useful PD model for the development of new therapeutic strategies.

## 1. Introduction

Parkinson’s disease (PD) is one of the most common neurodegenerative disorders, characterized by the progressive loss of dopamine (DA) neurons in the substantia nigra (SN) [1,2]. Manifestations of PD include motor and nonmotor symptoms, leading to a diminishment in independence, quality of life, and overall lifespans [3]. The accumulation and aggregation of α-synuclein (αSNCA) in the form of Lewy bodies (LBs) are associated with the dysfunctionality and deficiency of DA neurons [4]. Although the role of αSNCA aggregates in PD progression is quite definite, the temporal and pathophysiological relationships between αSNCA pathology, neurodegeneration, and clinical symptoms remain to be investigated [5]. A soluble presynaptic protein, αSNCA, is predominantly and ubiquitously expressed in the brain [4]. Substantial evidence suggests that αSNCA plays a central role in neurotransmitter release, most notably DA [6,7]. Under pathological conditions, certain αSNCA aggregates form LB-like inclusions in the brain, mediating synaptic dysfunction and reducing neurotransmitter release [8]. Abnormal αSNCA aggregates cause cytotoxicity, leading to neuronal injury and death [9,10]. It can be confirmed that PD progression highly correlates with the degree of neuronal cell loss.

Pathologic αSNCA from recombinant fusion protein could be internalized by cells and could eventually cause endogenous αSNCA LB-like pathology [11,12], which is the pathological hallmark of PD and is regarded as a surrogate marker for testing the efficacy of therapeutic approaches under development [13]. Researchers have explored overexpression of wild-type (WT) or mutant αSNCA in the formation of aggregates, the aim being to investigate the mechanisms associated with αSNCA-mediated neurotoxicity for potential pharmacological development [14,15]. However, the standard laboratory transfection procedure faces issues regarding the generalization of various in vivo applications. For example, viral vectors have previously achieved high efficiency for gene transfection into different cell types and animal models [16], but problems persist, including those related to toxicity, biosafety, and adverse immune responses. In contrast, non-viral delivery techniques have been associated with fewer safety issues, thus strengthening the likelihood that repeated administration is feasible [17]. Among the non-viral systems under development, lipoplexes have often exhibited highly efficient transfection for in vitro situations [17]. However, studies applying lipoplexes to various in vivo tissues have yielded inconsistent data [18,19]. An ideal model has yet to be established for early occurrence of disease phenotypes. In addition, neither viral nor non-viral approaches can be applied in the CNS noninvasively, owing to the blockade of the blood–brain barrier (BBB), which is another major issue in implementing in vivo CNS disease models in situ.

Research using focused ultrasound (US)-targeted microbubble (MB) destruction (UTMD) has made significant advances in non-viral and non-invasive gene transfection through transient cell membrane disruptions, allowing molecules to enter cells and enhancing the effect of UTMD-gene delivery [20,21]. Recently, studies have focused on using a combination of UTMD and nanoparticles to deliver drugs, genes and therapeutic compounds specific to the targeted sites [22]. We have previously shown that UTMD can focally and transiently open the BBB, allowing the deposit of macro-size therapeutic particles or genes on targeted CNS sites [23,24,25]. These findings support the prospect that UTMD can fulfill any specific gene delivery to CNS targets. We have recently succeeded in directly transfecting WT αSNCA into target sites to induce aberrant αSNCA aggregation (resulting in both an approximately two-fold increase in in vitro cell lines and a 1.68-fold increase in in vivo as compared to controls) [26]. These preliminary results demonstrated that artificially created αSNCA aggregates significantly reduced mitochondrial function. It is therefore reasonable to hypothesize that intravenous delivery of liposome-encapsulated αSNCA genes, when combined with UTMD, induces αSNCA-related PD motor symptoms and pathology.

Our aim in the present study is to develop a novel PD model via UTMD to achieve αSNCA-gene delivery into the CNS to artificially express αSNCA-related PD phenotype. The strategy is to deliver pαSNCA/liposome-encapsulated WT pαSNCA (LpαSNCA) into DA neurons in the SN. We examined the neuronal aggregates by using immunostaining. Moreover, by conducting biophysical and biochemical analyses, we identified consequences of the DA neuron deficit associated with the PD models. We compared both the disease progression and the phenotype expression in the UTMD-created PD model with the corresponding factors in conventional PD models, and to this end, we used neurotoxin MPTP and intracerebral (IC) injection of pαSNCA or LpαSNCA. According to our data, although PD pathology can be induced via different administration modalities, only UTMD can be used to repeatedly deliver pDNA genes into a target site in a safe and promising way.

## 2. Materials and Methods

### 2.1. Plasmid α-Synuclein (pαSNCA) and Liposomal pαSNCA (LpαSNCA) Preparation

The GFP-tagged WT αSNCA (NM_000345) plasmid construct (pαSNCA) was obtained (a gift from Dr. Guey-Jen Lee-Chen, National Taiwan Normal University). The amplified αSNCA cDNA was applying 5′-CCATGGATGGATGTATTCATGAAAGGAC-3′ forward and 5′-CCATGGCTTCAGGTTCGTAGTCTTG-3′ reverse primers and ligated into NcoI site of pEGFP-N1 (Takara Bio., San Jose, CA, USA) to produce the pαSNCA [26]. The pαSNCA encapsulating liposomes to construct liposomal pαSNCA (LpαSNCA) was prepared as previously reported [23,24,25]. The LpαSNCA was centrifuged and collected the supernatant. The concentration of pαSNCA encapsulated within the liposomes was determined at 260 nm using ND-1000 NanoDrop (Thermo Fisher Scientific Inc. Waltham, MA, USA). The particle sizes with a diameter of 85–125 nm and the zeta potential with approximately −50 mV were measured using Nano-ZS90 particle analyzer (Malvern Instruments, Malvern, UK). Cell transfection studies and characteristic properties were generated as described in Supplementary Materials.

### 2.2. Ethic Statement

All experiments involving animals were conducted according to the ethical policies and procedures approved by the ethics committee of the Institutional Animal Care and Use Committee, Chang Gung University, Taiwan (Approval No. CGU108-073). The investigation conforms NIH Guide in The Handbook of the Laboratory Animal Center for the Care and Use of Experimental Animals.

### 2.3. Animal Husbandry

C57BL/6 male mice were ordered from the National Laboratory Animal Center (Taipei, Taiwan). A total of 75 seven-week-old mice were used for the in vivo studies. Mice were housed in temperature- and humidity-controlled conditions (12/12 light/dark cycle, with the lights on from 8 p.m.–8 a.m.) and had free access to a standard diet and drinking water.

### 2.4. Ultrasound Systems

A 500-kHz single-element US transducer (diameter of 64 mm, focus length = 63.2 mm; Imasonics SAS, Besançon, France) with 69% of electric-to-acoustic efficiency was driven via a 33120A function generator (Keysight Technology, Palo Alto, CA, USA) connected with a 150A100B power amplifier (AR Inc., Souderton, PA, USA) and a Model 4421 power meter (Bird Electronics Corp., Cleveland, OH, USA) to generate the disruption sonication pulse. The input electrical power of 2.2 W (burst length = 10 ms, pulse-repetition frequency = 1 Hz, and sonication duration = 60 s) was applied (equivalent to negative pressure 0.5 MPa). Microbubbles (SonoVue^®^ average diameter = 2.5 μm, concentration = 5 × 10^8^ bubbles/mL; Bracco Diagnostics Inc., Milan, Italy) were injected intravenously at 0.2 mL/kg before focused US exposure.

### 2.5. In Vivo Transfection Studies and Characteristic Properties

In order to validate the effectiveness in vivo, three types of experimental procedures were used for this study, including neurotoxin intraperitoneal (IP) induction (MPTP), intracerebral (IC) convection (αSNCA expression), and intravenous (IV) infusion (αSNCA expression). Control mice received PBS only. A DA neuron-deficit PD mouse model induced by the neurotoxin MPTP was established previously [23,24]. Mice (*n* = 15) were subjected to an IP injection of 40 mg/kg MPTP-HCl (Aldrich-Sigma, St. Louis, MO, USA) once a day, 5 days a week for 3 weeks. IC injection was performed using a microinjection needle beveled in a 1 mm pulled glass capillary for intracellular-convection transfection. The sharpened needle was set to a syringe and connected to a microinfusion pump (Bioanalytical Systems, Lafayette, IN, USA). Then, this needle was implanted at anterior-posterior −0.5 mm from the bregma, lateral 2.5 mm from the midline, and ventral 3.5 mm from skull, using stereotaxic-guided fine needle. As large volume of infusion can induce IC hemorrhage, mice (*n* = 30) received the solution were infused by IC, at a flowrate at 1 μL/min to a volume of 5 μL of pαSNCA (pDNA at 2 μg/μL) or LpαSNCA (2 μg pDNA/μL) had been administrated for safety. In order to enhance expression in the IC-convention transfection, focused US exposure was further applied at the SN of the mouse brain. For IV infusion, transfection efficiency was analyzed for the LpαSNCA at three different doses (pDNA at 10 μg, 40 μg and 60 μg, respectively) with and without UTMD. The pDNA doses used were based on the previous studies for UTMD-gene transfer [27,28]. The injected dose of MBs was 0.2 mL/kg and the duration of focused US exposure was 60 s. Afterward, each group (*n* = 30) was run once a week for 3 weeks. On day 22, animals were sacrificed, and brain tissues were collected and stored at −80 °C for further investigations. All assays included the control group.

### 2.6. In Vivo Imaging System (IVIS)

The IVIS instrument (IVIS-200, Xenogen Corporation, Alameda, CA, USA) was used to identify the transfection location of gene delivery. The images were acquired after IV transfection, without or with UTMD, to analyze the signal intensity, in order to determine the UTMD-induced gene transfection activity in the brains. Animals were placed and analyzed for fluorescence intensity. GFP was excited at 488 nm (filter range 445 to 490) and detected at 510 nm. Mice were scanned at 1, 2 and 5 days, respectively, with constant exposure time for all the groups. Data was performed as intensity signal photons/s/cm^2^ using the Living Image 2.5 software (Caliper Life Sciences, Hopkinton, MA, USA).

### 2.7. Neurochemical Analysis by HPLC

Concentrations of striatal DA, DOPAC, and HVA in cleared extracts obtained by homogenization of each tissue sample in 500 μL of protein extraction solution (PRO-PREP^TM^, iNtRON Biotechnology Inc., Summit, NJ, USA). The homogenized solutions were centrifuged for 30 min and HPLC system was used to analyze the soluble fraction as previously reported [23,24].

### 2.8. Assessment of Motor Function

The rotarod apparatus from Singa Technology Corp. (Taipei, Taiwan) was used to evaluate animal motor coordination and balance assessments measured by the latency to fall from the apparatus. We placed the mice on a rotarod apparatus accelerating from 5 to 30 rpm in 5 min. Each mouse was executed for three repeated trials at each time point, with 15 min rest between two trials. The latency to fall was recorded when the mouse fell off the rod. Repeated testing was performed twice a week over a 3-week period. The latency to fall was normalized to the control as presented in percentage.

### 2.9. Immunohistochemistry (IHC)

Brain tissues were sectioned on a standard rotary microtome (Leica, Berlin, Germany). Tissue sections were collected and counterstained with hematoxylin-eosin (HE). Then, the sections were subjected to staining with the primary antibodies. Primary antibodies were tyrosine hydroxylase (TH, 1:1000) (Santa Cruz Biotech., Dallas, TX, USA), P-CREB (1:500, Upstate Comp., Lake Placid, NY, USA), caspase 3 (1:500), or MDA (1:100). After raining with PBS, sections were stained with 3,3′-diaminobenzidine (DAB) as described [24]. Subsequently, the selected sections were further performed by using Tuj1 (1:500), GFAP (1:500), and IBA1 antibody (1:200, Santa Cruz Biotech., Dallas, TX, USA). The stained sections were counterstained with DAPI, mounted and examined microscopically.

### 2.10. Statistical Analysis

We used the one-way ANOVA Tukey’s comparison test or nonparametric Mann–Whitney U test to compare the statistical significance of the difference between groups, where appropriate. *p* < 0.05 was used as the criterion for significance. Statistically significant difference was analyzed using version 26.0, SPSS (SPSS Inc., Chicago, IL, USA).

## 3. Results

### 3.1. UTMD-Enhanced Gene Transfection on Targeted Regions for the Generation of DA Neuron-Deficit PD Models

**Figure 1:**
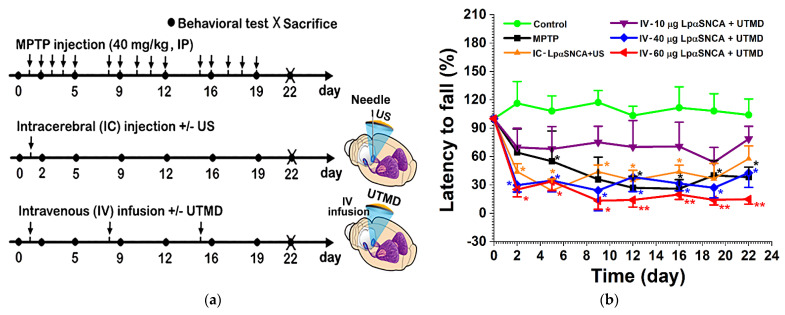
(**a**) Timeline of study design and experimental scheme. Arrows point to when intraperitoneal injections of MPTP (40 mg/kg) were given once a day, 5 days per week for 3 weeks; when single intracerebral (IC) injections of pαSNCA or LpαSNCA +/− US were given; when intravenous (IV) infusions of LpαSNCA +/− UTMD were given; and when animals were scheduled for behavioral testing and sacrificed; (**b**) motor performance of the various groups of mice trained with a rotarod apparatus. Latency to fall for each mouse was recorded on the rotarod performance. Statistical difference was performed with the Mann–Whitney U test; * *p* < 0.05; ** *p* < 0.01; *n* ≥ 5 for each group. MPTP = 1-methyl-4-phenyl-1,2,3,6-tetrahydropyridine; US = focused ultrasound; UTMD = focused ultrasound-targeted microbubble destruction; LpαSNCA = liposomal plasmid α-synuclein.

For the current study, we developed three types of experimental procedures to induce different DA neuron-deficit PD models in parallel. The detailed time course for the intraperitoneal (IP) MPTP-treatment, the intracerebral (IC) injection of pαSNCA or LpαSNCA with or without (+/−) US (IC-pαSNCA or LpαSNCA +/− US), and the intravenous (IV) infusion of LpαSNCA with or without UTMD (IV-LpαSNCA +/− UTMD), scheduled rotarod performance, and animal sacrifice was presented (Figure 1a).

Next, we set out to determine whether or not, in general, the aggregation of transfected αSNCA, the loss of DA neurons, and increased neuroinflammation play a role in the fundamental pathogenic processes underlying motor symptoms during disease progression. Thus, we examined motor deficits longitudinally with a rotarod device (Figure 1b). From day 2 to day 22, we observed remarkable motor dysfunction in the IV-60 μg LpαSNCA (pαSNCA at 60 μg) + UTMD group. Three weeks after IV infusion with 60 μg LpαSNCA and UTMD, this group exhibited an approximately 90% reduction in latency to fall compared to the control group (11.7 ± 10.21% vs. 98.7 ± 6.11%). Obviously, the IV-10 μg LpαSNCA (pαSNCA at 10 μg) + UTMD group exhibited insignificant motor deficit when compared with the control group. Of note, the MPTP-treatment group, the IC-LpαSNCA + US group, the IV-40 μg LpαSNCA (pαSNCA at 40 μg) + UTMD group, and the IV-60 μg LpαSNCA + UTMD group exhibited significantly more impairment in motor performance than was the case in the control group (** *p* < 0.01; * *p* < 0.05). It is also noteworthy that only the IV-40 μg LpαSNCA + UTMD group and the IV-60 μg LpαSNCA + UTMD group exhibited significantly impaired performances at all time-points from day 2 to day 22; the MPTP-treatment group and the IC-LpαSNCA + US group displayed impaired motor function only at some time-points. These results indicate that UTMD-stimulated αSNCA overexpression induced the most severe motor deficits, although mice in the MPTP-treated group and the IC-LpαSNCA + US group also presented significant impaired motor function.

**Figure 2:**
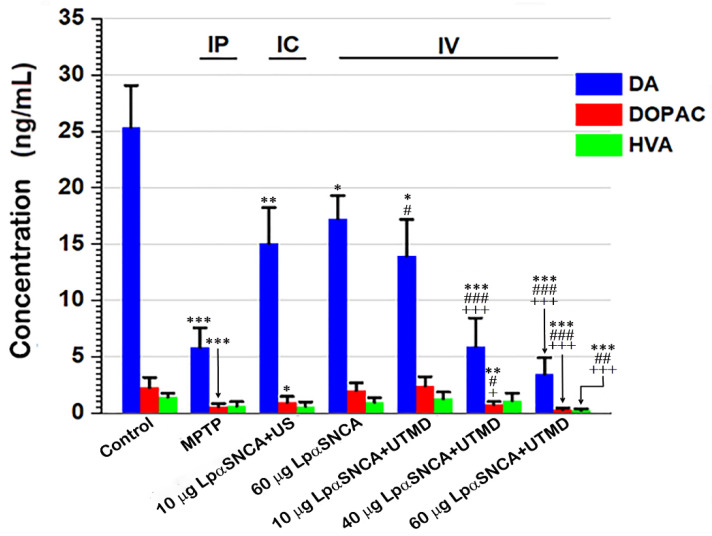
The neurotransmitter level of DA and its metabolites: DOPAC and HVA. Bar graphs represent the mean ± standard deviation, and statistical analyses were performed via ANOVA followed by Tukey’s post hoc test. *, ** and *** indicate *p* < 0.05, *p* < 0.01 and *p* < 0.005 vs. the control group; ^#^, ^##^ and ^###^ indicate *p* < 0.05, *p* < 0.01 and *p* < 0.005 vs. the IC-LpαSNCA + US group; ^+^ and ^+++^ indicate *p* < 0.05 and *p* < 0.005 vs. the IV-LpαSNCA group; *n* ≥ 5 for each group. MPTP = 1-methyl-4-phenyl-1,2,3,6-tetrahydropyridine; US = focused ultrasound; UTMD = focused ultrasound-targeted microbubble destruction; LpαSNCA = liposomal plasmid α-synuclein. IP = intraperitoneal; IC = intracerebral; IV = intravenous; DA = dopamine; DOPAC = 3,4-dihydroxyphenylacetic acid; HVA = homovanillic acid.

To analyze the functional consequences of SN brains using different experimental procedures for inducing DA neuron-deficit PD models, we used a set of quantitative HPLC. With this set, we determined the neurotransmitter levels of DA and its two metabolites: DOPAC and HVA (Figure 2). Our neurochemical analysis revealed a highly significant decrease in DA (≥70%) in MPTP-treated mice, while the greatest decline in DA (≥85%) took place in the IV-60 μg LpαSNCA group with UTMD-treated mice. Statistically, UTMD-treated mice with the IV-40 μg LpαSNCA or IV-60 μg LpαSNCA exhibited significantly lower DA than did the control mice (*** *p* < 0.005), the IC-LpαSNCA + US mice (^###^ *p* < 0.005), and the IV-60 μg LpαSNCA mice (^+++^ *p* < 0.005). The reduction in DOPAC was also significantly higher in UTMD-treated mice with the IV-40 μg LpαSNCA or the IV-60 μg LpαSNCA than in the control-group mice (*** *p* < 0.005 and ** *p* < 0.01), and the IC-LpαSNCA + US mice (^###^ *p* < 0.005 and ^#^ *p* < 0.05), and the IV-60 μg LpαSNCA mice (^+++^ *p* < 0.005 and ^+^ *p* < 0.05). Of note is that the HVA level in the UTMD-treated mice with the IV-60 μg LpαSNCA was significantly lower than in the other groups (*** *p* < 0.005 vs. the control group, ^##^ *p* < 0.01 vs. the IC-LpαSNCA + US group, ^+++^ *p* < 0.005 vs. the IV-60 μg LpαSNCA group). Although both the DA levels and the DOPAC levels in the MPTP-induced PD model were relatively low, the levels of metabolite HVA did not significantly differ from those in the control group. These results point to a particular trend: both DA and its metabolites decreased in the UTMD-treated mice depending on the administration dose, and the decreases reached significant levels for all DA and its metabolites only at a 60 μg dose. The results suggest that UTMD-gene transfection is the most efficient strategy for the establishment of DA neuron-deficit phenotype.

### 3.2. Protein Expression in DA Neuron-Deficit PD Models

**Figure 3:**
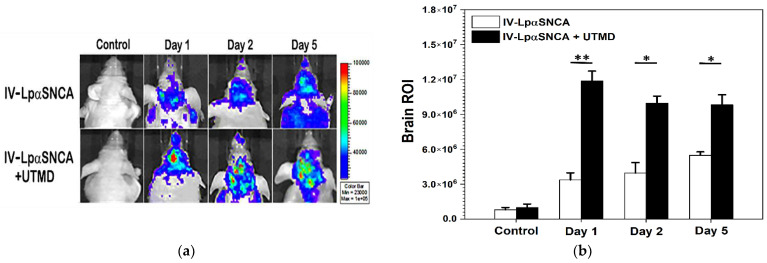
(**a**) Representative peak bioluminescent imaging acquired on day 1, day 2, and day 5 after LpαSNCA IV infusion +/− US exposure in the presence of microbubbles (IV-LpαSNCA vs. IV-LpαSNCA + UTMD). Transfection efficiency was commenced 1 day after gene transfection; (**b**) Data represent an average photo flux from all injection sites in the group (p/s/cm^2^/sr) ± SD. * *p* < 0.05; ** *p* < 0.01 (Mann–Whitney U test); *n* = 3 for each group. IV = intravenous; UTMD = focused ultrasound-targeted microbubble destruction; LpαSNCA = liposomal plasmid α-synuclein; ROI = region of interest.

To assess the performance of UTMD-induced gene transfection in the brain after IV-infusion of LpαSNCA, we evaluated the efficacy of targeted transfection by conducting IVIS observation on day 1, day 2 and day 5 (Figure 3a). Based on our previous studies, we administered a nominal and effective dose of 40 μg of LpαSNCA in order to distinguish the groups from one another regarding UTMD-gene transfection [23,27]. UTMD-triggered IV-LpαSNCA delivery demonstrated a significantly greater elevation in fluorescence expression than was the case with IV-LpαSNCA alone (** *p* < 0.01, * *p* < 0.05) (Figure 3b). Our imaging confirms the successful transfection of pαSNCA into the targeted brain region.

**Figure 4:**
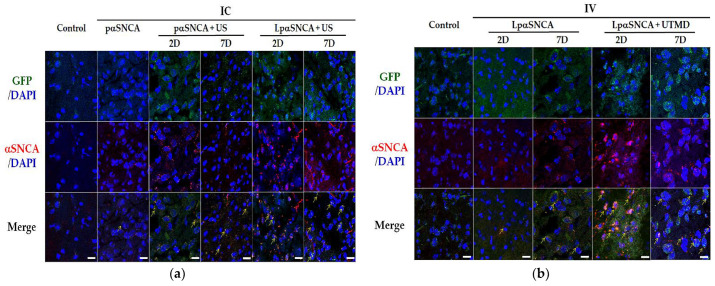
Representative intracellular aggregation of mice following (**a**) IC with naked pαSNCA (10 μg), LpαSNCA (10 μg pDNA) or LpαSNCA (10 μg pDNA) +/− US expressed at day 2 and day 7, and (**b**) IV with LpαDNA (60 μg pDNA) +/− UTMD expressed on day 2 and day 7. *n* ≥ 3 for each group. IC = intracerebral; IV = intravenous; US = focused ultrasound; UTMD = focused ultrasound-targeted microbubble destruction; LpαSNCA = liposomal plasmid α-synuclein; D = day. Arrows indicate aggregates. Scale bars = 20 μm.

In our previous studies, we established and evaluated the neurotoxin IP MPTP-induced DA neuron-deficit model [23,24]. In the present study, we are evaluating the effect that IC +/− US and IV +/− UTMD has on the efficiency of pαSNCA transfection in mouse SN (Figure 4). Because the transfection efficiency of the IV-60 μg LpαSNCA + UTMD group generated the most prominent phenotype, it was under this dose that we compared IC and IV αSNCA administration conditions with one another regarding intracellular aggregation induced by transgene transfection. In IC groups (Figure 4a), the US exposure on brain tissues could induce relatively high fluorescent signals (green arrows). On day 2 and day 7, greater levels of αSNCA and GFP proteins were co-expressed in the IC-LpαSNCA + US group than in the control group, the IC-pαSNCA group, and the IC-pαSNCA + US group. The IV-LpαSNCA + UTMD group (Figure 4b) exhibited even higher fluorescent levels than the IV-LpαSNCA-only group, a result indicating that LpαSNCA deposition into the SN was enhanced via UTMD and eventually induced αSNCA and GFP expression. Additionally, strong GFP fluorescence colocalized with intracellular αSNCA aggregates was noted on day 2, and was even stronger 7 days after treatment in both the IC-LpαSNCA + US group and the IV-60μg LpαSNCA + UTMD group. Although our in vitro research using UTMD has addressed high transfection efficiency with naked pαSNCA (Supplementary Figures S1 and S2), the findings reveal that the use of LpαSNCA can protect plasmid DNA from degradation and can thus increase gene expression in vivo. The “Supporting Information” presents intracellular aggregation regarding αSNCA and GFP protein expression at day 2 and day 7 of IC and IV. (Supplementary Figure S3).

### 3.3. Neuronal Loss in SN

**Figure 5:**
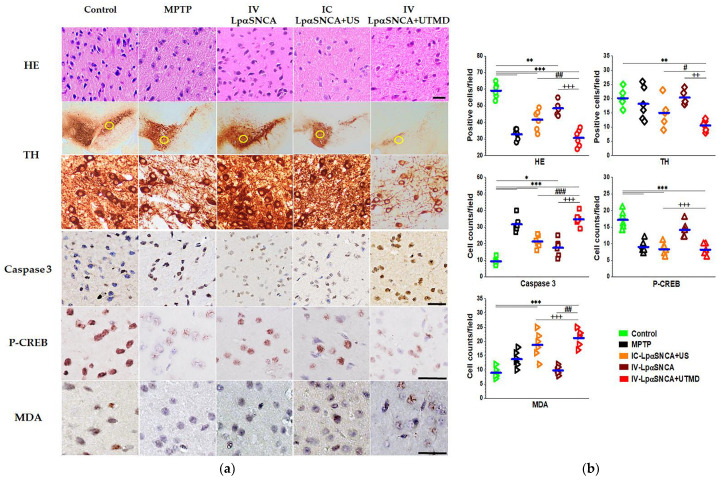
(**a**) Microscopic observation of histological damage, HE staining, TH, caspase 3, P-CREB, and MDA numbers with the control and PD groups (MPTP, IC with LpαSNCA (10 μg pDNA), and IV with LpαDNA (60 μg pDNA) +/− UTMD). TH immunostaining of the control and PD groups (magnification, ×40; high magnification, ×400). Scale bars = 100 μm. (**b**) Bar graphs represent the mean ± standard deviation, and statistical analysis was performed via ANOVA with the post hoc Tukey test. *, **, and *** indicate *p* < 0.05, *p* < 0.01, and *p* < 0.005 vs. the control group; ^#^, ^##^, and ^###^ indicate *p* < 0.05, *p* < 0.01, and *p* < 0.005 vs. the IC-LpαSNCA + US group; ^++^ and ^+++^ indicate *p* < 0.01 and *p* < 0.005 vs. the IV-LpαSNCA group; *n* ≥ 3 for each group. MPTP = 1-methyl-4-phenyl-1,2,3,6-tetrahydropyridine; IC = intracerebral; IV = intravenous; US = focused ultrasound; UTMD = focused ultrasound-targeted microbubble destruction.

We assessed histological changes and performed a quantitative summary of positive cell counts in the SN (Figure 5). The HE staining demonstrated that the SN neurons were significantly high in number and density with clear nuclear structures in the control. As shown in Figure 5a (top row), DA neuron-deficit PD brains were significantly lower in number and density of condensation neurons than was the case with the control group (Figure 5b, top row) (** *p* < 0.01, *** *p* < 0.005). Low- and high-power photomicrographs demonstrate DA neuron loss using anti-TH staining (Figure 5a, 2nd and 3rd rows). Staining results show that the loss of DA neurons (approximately 25%) in the MPTP-treated brain was greater than in the control group. Of note, the IV-60 μg LpαSNCA + UTMD group exhibited the most significant damage to TH-positive neurons with a 50% decrease in cell number (Figure 5b, top row, ** *p* < 0.01 vs. the control). The IV-60 μg LpαSNCA + UTMD group also exhibited a significantly lower TH-positive cell count than in the IC-LpαSNCA + US and IV-LpαSNCA groups. These results show that the IV-60 μg LpαSNCA + UTMD group could generate the most significant loss of TH-positive cells.

We also determined the levels of caspase 3, P-CREB, and MDA to evaluate the effects of DA neuron-deficit PD models (Figure 5a, 4th to 6th rows). To analyze the degree of cellular apoptosis, we quantitated caspase 3 staining in the nucleus and cytoplasm. In all DA neuron-deficit PD groups, caspase 3 in the SN was significantly greater than in the control group (Figure 5b, 2nd row, *** *p* < 0.005; * *p* < 0.05). Of all the study groups, the IV-60 μg LpαSNCA + UTMD group exhibited the highest cellular-apoptosis level (^###^ *p* < 0.005 vs. the IC-LpαSNCA + US group; ^+++^ *p* < 0.005 vs. the IV-LpαSNCA group). To further investigate the neuronal survival in DA neuron-deficit PD models, we measured P-CREB as the neuronal marker. The IV-60 μg LpαSNCA + UTMD group exhibited both a greater increase in αSNCA protein expression in the SN and a significantly greater reduction in neuronal survival than was the case in the other groups (Figure 5b, 2nd row, *** *p* < 0.005 vs. the control; ^+++^ *p* < 0.005 vs. the IV-LpαSNCA group). In addition, MDA levels in SN, indicating oxidative stress, were higher in the IV-LpαSNCA + UTMD group than in the other groups (Figure 5b, 3rd row, *** *p* < 0.005 vs. the control; ^###^ *p* < 0.005 vs. the IC-LpαSNCA + US group; ^+++^ *p* < 0.005 vs. the IV-LpαSNCA group).

### 3.4. Pathological Changes in SN

**Figure 6:**
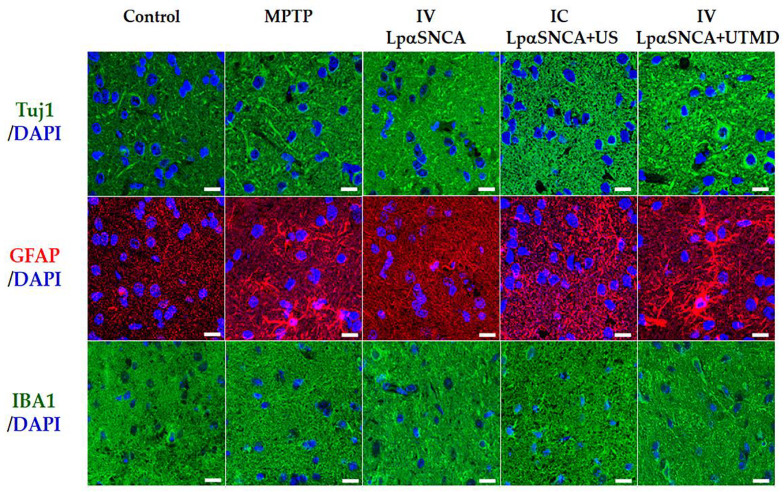
Pathological changes in the substantia nigra (SN) of different DA neuron-deficit PD models (MPTP, IC with LpαSNCA (10 μg pDNA), and IV with LpαDNA (60 μg pDNA) +/− UTMD). Images of fluorescence-labeled IHC SN-based staining (green: Tuj1 and IBA1 to identify neurons and microglia; red: GFAP to identify astroglia; blue: DAPI to mark cell nuclei). *n* ≥ 3 for each group MPTP = 1-methyl-4-phenyl-1,2,3,6-tetrahydropyridine; IC = intracerebral; IV = intravenous; US = focused ultrasound; UTMD = focused ultrasound-targeted microbubble destruction. Scale bars = 20 μm.

To better visualize the pathological changes in the DA neuron-deficit PD models, we used fluorescence-labeled IHC staining (green: Tuj1 and IBA1 to identify neurons and microglia; red: GFAP to identify astroglia; blue: DAPI to mark cell nuclei). Consequently, we identified neurotoxicity effects in SN (Figure 6). In contrast to the control brain, DA neuron-deficit PD brains displayed deficits in their neurite outgrowth (top row) with decreased branching, lengths, and Tuj1 expression. The SN brains associated with the IP-MPTP-treatment, IC-LpαSNCA + US, and IV-60 μg LpαSNCA + UTMD exhibited greater levels of GFAP (middle row) and a greater shift to microglia morphology (bottom row) than did the control group. Of note, the astroglia and microglia of the IV-60 μg LpαSNCA + UTMD brains exhibited a more ameboid morphology than did the astroglia and microglia of the brains in the other groups, which is suggestive of an activated state. IHC evidence strongly supports that UTMD enhanced αSNCA expression, resulting in neural toxicity stemming from increased oxidative stress, increased neuroinflammation, increased apoptosis, and eventually induced DA loss, and thus coincides with PD phenotype pathology.

## 4. Discussion

Three different administration routes to induce PD mouse models in this study: MPTP IP injections, pαSNCA IC injections, and pαSNCA IV administrations + UTMD. The MPTP is a well-recognized PD model and is to serve as a reference to benchmark PD pathogenesis phenotype when compared to αSNCA transduction. However, rather than IC injecting the pαSNCA, the aim of this study is to propose and validate an alternative way to transduce pαSNCA to present PD pathogenesis via IV administration, in conjunction with UTMD. This novel approach provides advantages including less invasiveness and repeated triggering flexibility to benefit stable αSNCA-based PD model induction.

Here, we have successfully demonstrated that UTMD can induce the expression of full-length αSNCA-GFP recombinant fusion proteins to present αSNCA aggregation, neurotoxicity, and loss of DA neurons. We reported that UTMD-induced αSNCA expression can subsequently induce αSNCA aggregation and dopaminergic neuron loss in mouse SN to mimic PD phenotype. We believe that the key novelty of this study is its development of a novel US-based gene-delivery technology to establish in vivo PD models, which can efficiently express PD-related phenotype and neuropathological features.

Therapeutic US applications have expanded to include most major organ sites [29,30], and UTMD can temporarily disrupt the BBB [24]. Previous studies employed other gene transfection modalities to overexpress αSNCA and form aggregation, leading to neuronal apoptosis in cell models of α-synucleinopathy [31,32]. However, generating animal PD models with progressive αSNCA aggregation and DA neuronal loss has been viewed as challenging. Researchers have used several strategies to create animal PD models. For example, MPTP-induced mouse models have facilitated investigations into PD pathogenesis and therapeutics, but these models have hardly any aggregate formation in brains, and DA neuron susceptibility to MPTP is not consistent among mouse strains [33]. To generate PD models, researchers have adopted transgenic overexpression of αSNCA in animals by directly injecting soluble αSNCA oligomers or fibrils or αSNCA-viral vector into the striatum or SN of animals [34]. However, no study has examined overexpression of αSNCA using UTMD technology to establish an animal model. Because αSNCA aggregation-induced neurotoxicity plays a critical role in dopaminergic neuronal loss, the misfolded protein aggregation may enhance disease-related phenotype. In this study, we have shown UTMD-BBB opening induced αSNCA overexpression, as a way to mimic the αSNCA-related PD phenotype, and offers several advantages, including its noninvasive procedure, dose-dependent controls, phenotype promoting stability and repeatability, and fast phenotype expression. It is ideal to show if IC injection with two other higher doses of pαSNCA produce better or similar results with IV pαSNCA + UTMD. However, the experiments were not performed in our study due to the safety concern, as mentioned in Section 2.5. Therefore, our study results cannot conclude that IV pαSNCA + UTMD is more effective in terms of inducing expression of pαSNCA, compared with IC injection of pαSNCA. Nevertheless, our study suggests that IV pαSNCA + UTMD is an efficient and simplified method to induce PD mouse model.

In our current in vivo study, low-intensity US irradiation with MBs temporarily opened the BBB to permit drug/gene delivery to the targeted brain region [35,36]. We also confirmed that the combined use of focused US and MBs can effectively permeate the BBB to overexpress αSNCA in the SN region. So that US-stimulated transfection can be successfully induced in vivo, US parameters must be carefully selected and controlled. These parameters include the selection of MB dosage, US exposure (e.g., frequency, pressure, and duration), and the selection of plasmid concentrations. In this study, to aim US energy into the SN regions of small animals, we used focused US transducer for our in vivo PD studies. Drawing on our previous successful research on various types of cells, we selected 2.2 W of electrical power for brain cells [24,25]. These conditions successfully opened the BBB in vivo [23,24,25] and constitute a feasible approach to efficient gene transfection, which in turn can help model specific phenotypes of α-synucleinopathy.

The major difference between our current work and our previous work concerns our current decision to produce neurotoxic αSNCA aggregates in animals by using the UTMD approach. We determined the toxicity of αSNCA aggregation in brain SN neurons. In in vivo results show that UTMD-mediated αSNCA aggregation successfully induces pathological changes. Caspase 3 activation exhibits substantial neuronal apoptosis, which usually leads to neuronal death [37]. We examined the apoptotic marker caspase 3 and the oxidative stress marker MDA to determine the neurotoxicity induced by UTMD-mediated αSNCA expression. P-CREB exhibits neuronal plasticity and cell survival [2,38]. We have showed that CREB phosphorylation was perturbed in our UTMD-mediated PD-like mouse model. The reduction in P-CREB was associated with impaired neurite outgrowth, as shown by Tuj1 staining. Increased GFAP and IBA1 immunoreactivity pointed to an activated neuroinflammatory SN-based response, which may be attributable to αSNCA aggregation. Neuroinflammation and increased oxidative stress may therefore cause apoptosis and finally the loss of DA neurons in SN. All of these findings suggest that αSNCA expression and aggregation via UTMD-mediated αSNCA expression may reduce neurite outgrowth, increase oxidative stress, and activate neuroinflammation, apoptosis and neuronal loss.

## 5. Conclusions

In our current study, we have demonstrated that our PD models, by using UTMD CNS gene delivery, can account for disease phenotype in ways that permit the effective delivery of αSNCA gene plasmid (pαSNCA) into DA neurons into SN. The pαSNCA transfection mediated by UTMD was equally efficient in vivo experiments. In gene transfection, US sonication with MBs enhances gene transfection, which is the key factor in generating gene-overexpressing disease models. In conclusion, UTMD effectively induces clinically relevant PD pathogenesis and can benefit the development of new PD therapeutic strategies.

## Data Availability

The data presented in this study are within the article and Supplementary Materials, or on request from the corresponding authors.

## Supplementary Materials

The following supporting information can be downloaded at: https://www.mdpi.com/article/10.3390/pharmaceutics14020444/s1, Figure S1: (A) Generation of αSNCA-GFP-expressing N2A. Combination of GFP fluorescence (green) and indirect immunofluorescence for αSNCA (red). Overlapped signals are yellow or orange. Scale bars = 10 μm. (B) CyroEM images of αSNCA fibril morphology. (C) Immunoblots detecting αSNCA and GFP expression levels for different experimental groups. There was significant difference according to the Mann–Whitney U test, ** *p* < 0.01. pαSNCA = plasmid α-synuclein; MBs = microbubbles; UTMD = ultrasound-targeted microbubble destruction, Figure S2: (A) Expression levels of Caspase 3, MDA, BCL2, and BAX in transfected N2A cells in the experimental groups. Caspase 3, MDA, BCL2, and BAX (red); GFP (green); DAPI (the nucleus, blue). (B) Effects of transfection efficiency in N2A cells. Fluorescence images showing the localization of Tuj1 immunoreactivity (green), GFP-expressing cells (green), GFAP immunoreactivity (red), and DAPI (blue). Statistically significant difference was performed with the Mann–Whitney U test; ** *p* < 0.01. Scale bars = 10 μm, Figure S3: Representative protein expression following IC with naked pαSNCA, LpαSNCA or LpαSNCA +/− US expressed at day 2 and day 7, and IV with LpαDNA +/− UTMD expressed on day 2 and day 7. Relative intensity compared to the control. * *p* < 0.05; ** *p* < 0.01 (Mann–Whitney U test). US = focused ultrasound; UTMD = focused ultrasound-targeted microbubble destruction; ROI = region of interest; IC = intracerebral; IV = intravenous; D = day. Arrows indicate aggregates. Scale bars = 10 μm.
