# Supplementary material for: Focused Ultrasound-Induced Blood–Brain Barrier Opening Enhanced α-Synuclein Expression in Mice for Modeling Parkinson’s Disease
Source: Pharmaceutics. 2022 Feb 18;14(2):444. doi: 10.3390/pharmaceutics14020444 (PMC8876143; doi:10.3390/pharmaceutics14020444)
Supplement: Supplementary file 1 [file pharmaceutics-14-00444-s001.zip › pharmaceutics-1567888-supplementary.pdf]

pharmaceutics

Supplementary Materials

Focused Ultrasound-Induced Blood–Brain Barrier Opening Enhanced α-Synuclein Expression in Mice for Modeling Parkinson's Disease

Chung-Yin Lin [1,2], Ching-Yun Huang [1], Chiung-Mei Chen [3,*] and Hao-Li Liu [4,5,*]

1 Medical Imaging Research Center, Institute for Radiological Research, Chang Gung University, Taoyuan 33302, Taiwan; winwood7@mail.cgu.edu.tw (C.-Y.L.); sun01415@mail.cgu.edu.tw (C.-Y.H.)
2 Department of Nephrology and Clinical Position Center, Chang Gung Memorial Hospital, Taoyuan 33302, Taiwan
3 Department of Neurology, Chang Gung Memorial Hospital, College of Medicine, Chang Gung University, Taoyuan 33302, Taiwan
4 Department of Electrical Engineering, National Taiwan University, Taipei 10617, Taiwan
5 Department of Biomedical Engineering, National Taiwan University, Taipei 10617, Taiwan
* Correspondence: cmchen@cgmh.org.tw (C.-M.C.); hlliu@ntu.edu.tw (H.-L.L.); Tel.: +886-3-3281200 ext. 8729 (C.-M.C.) or +886-2-33665416 (H.-L.L.)

Supplementary Materials and Methods

Cell Culture

The Neuro2a (N2A) cells were from ATCC (American Type Culture Collection, Rockville, MD, USA). Cells were grown in 75 cm^2 cell culturing flasks and 6-wells plates with a density of 2×10^5 per well in the DMEM medium at 37 °C with 5 % CO_2 incubator. A hemocytometer and the trypan blue assay were used to examine cell viability. Only cells with initial viability exceeding 80% were used.

Ultrasound Sonicator

An ultrasonic transducer with a frequency of 20 kHz, output power density of 1.25 W/cm^2, and duty cycle of 100% (3 mm tip, VCX750, Sonics and Materials, Newton, CT, USA) was used in vitro to produce uniform ultrasound field distribution in a cultured cell dish.

In Vitro Transfection Studies and Characteristic Properties

The N2A was used to test gene transfer efficiency by the effect of insonation. Four groups were tested (n = 6 per group): 1) no-transfection control (no plasmid, no MBs), 2) the pαSNCA only; 3) the pαSNCA with MBs (no US); 4) pαSNCA with MBs and US. The pαSNCA was pipetted to the wells in a concentration of 2 μg/mL aliquots. Cells were placed at incubator prior application of US exposure. 12.5 μL of MBs were added to the experimental groups 3 and 4. The ultrasound sonication was generated to an intensity of about 1.25 W/cm^2 for 2 s for experimental group 4. The media were replaced from all the wells to remove any un-incorporated plasmids or MBs after sonication. Cells were then grown for 48 h after pαSNCA was delivered. After rinsing in PBS, an anti-αSNCA antibody was used to determine whether the aggregated SNCA had been expressed after plasmid transfection with sonoporation. These cells were then observed and photographed using inverted fluorescence microscopy (ZEISS LSM780, Carl Zeiss MicroImaging GmbH, Jena, Germany) to visualize the intracellular αSNCA and GFP co-expression and aggregates in N2A cells. Experiments were carried out at least three independent assays included the control cells.

Fibril Preparation and Morphological Structure

The following groups, including the control, the pαSNCA, and the pαSNCA+UTMD, were prepared. Fibrils have been prepared according to previous studies [1,2]. Briefly, the cells were collected and lysed with a probe sonicator for 10 min in an iced bath. The lysed cell solution was centrifuged and the soluble fraction was collected into a fresh tube. The tube was then heated in boiling water for 10 min and titrated with HCl at pH 4.5 to remove the undesirable precipitants. The supernatant solution was concentrated and purified by column chromatography using 20 mM Tris-HCl buffer as eluent to obtain a colorless solution. 3 μL of the purified solutions from each sample were pipetted on the 200 mesh copper grids, plunged into liquid ethane, stored in liquid nitrogen for later use. Afterward, samples were imaged using a JEM-2100 Plus electron microscope (Jeol Ltd, Tokyo, Japan) operated at 100 kV with a LaB_6 source. Digital micrographs were recorded with a 2048 × 2048 Gatan OneView CCD camera.

Western Blotting

Protein lysates were prepared from cells. Briefly, the cultured cells were lysed in protein extraction buffer, and then centrifuged for 30 min at cold room. After harvest the top layer, the protein concentrations were quantified, loaded on to a 10 % SDS-PAGE gel, and transferred to a PVDF membrane. We incubated the membranes with blocking solution, containing a rat anti-SNCA antibody (1:200) or a rabbit anti-GFP antibody (1:2000) (Proteintech., Chicago, IL, USA) overnight followed by the secondary mouse anti-rat antibody (1:1000) (Molecular Probes Inc., Grand Island, NY, USA) for 1 h. Rabbit anti-βactin (1:5000) (BD Biosci., San Jose, CA, USA) was employed as an experimental internal control. Afterward, the quantitative analysis of the western blot was detected using Amersham ECL reagents (Little Chalfont, UK).

Immunocytochemistry (ICC)

ICC analysis of caspase 3 (1:1000), malondialdehyde (MDA, 1:200), BCL2 (1:200), BAX (1:1000) (Abcam, Cambridge, MA, USA), Tuj1 (1:1000) and GFAP (1:200) (Santa Cruz Biotech., Dallas, TX, USA) were performed on N2A cells. Following fixation in 4% paraformaldehyde, cells were incubated with the primary antibodies as described above. Cells were washed several times in PBS and incubated for 1 h with secondary antibodies. Secondary antibodies were rabbit anti-mouse fluorescence 488 (1:1000) and donkey anti-mouse fluorescence 594 (1:1000) (Molecular Probes Inc., Grand Island, NY, USA). Coverslips were mounted using an antifade mounting medium with the nuclear marker DAPI (Vector Lab., Burlingame, CA, USA). For confocal microscopy a Leica TCS SP8X confocal microscope (Wetzlar, Germany) was used. 488 nm and 594 nm laser excitation wavelengths were utilized.

Immunoblotting

Protein lysates were prepared from brain tissues for immunoblotting analysis. Briefly, the brain tissues from each group were lysed in protein extraction buffer, and then centrifuged for 30 min at cold room. After harvest the top layer, the protein concentrations were quantified, loaded on to a 10% SDS-PAGE gel, and transferred to a PVDF membrane. We incubated the membranes with blocking solution, containing a rat anti-SNCA antibody (1:200) or a rabbit anti-GFP antibody (1:2000) (Proteintech., Chicago, IL, USA) overnight followed by the secondary mouse anti-rat antibody (1:1000) (Molecular Probes Inc., Grand Island, NY, USA) for 1 h. Rabbit anti-βactin (1:5000) (BD Biosci., San Jose, CA, USA) was employed as an experimental internal control. Afterward, the quantitative analysis of the western blot was detected using Amersham ECL reagents (Little Chalfont, UK).

Supplementary Results

UTMD-Stimulated pαSNCA Expression In Vitro

To evaluate the sonoporation potential for stimulating gene expression as a transfection tool, we prepared pαSNCA-GFP constructs for the assessment of in vitro transfection efficiency 48 h post-transfection. Transfected cells showed that fluorescent expression of αSNCA (red) and GFP (green) proteins resulted in the formation of puncta or aggregates after UTMD (Figure S1A). Without US, neither pαSNCA nor pαSNCA plus MBs can induce gene expression. In order to demonstrate whether UTMD affects αSNCA aggregation, we further characterized fibrils by using cryoEM (Figure S1B). The cellular extracted fibrils formed by αSNCA were straight and between 20 and 300 nm long. Although αSNCA filaments in the absence of sonication were imaged, they were hardly detectable by means of cryoEM. This result suggests that αSNCA fibril formation is not favorable without powerful hydro-mechanical shear forces. The fibril-formation rate was higher in the presence of UTMD than other conditions. Using immunoblotting, we quantitated the αSNCA-GFP protein expression (Figure S1C). A significant band appeared at about 49 kDa, and its intensity value for UTMD-mediated gene expression was about 6-fold higher than in other conditions (control, pαSNCA, and pαSNCA+MBs). We can conclude that αSNCA-GFP expression was significantly higher in the UTMD context (pαSNCA+UTMD group, $^{**}p < 0.01$) than in the other groups, indicating that MBs combined with US exposure can act as seeds capable of initiating and accelerating αSNCA fibril or aggregation formation.

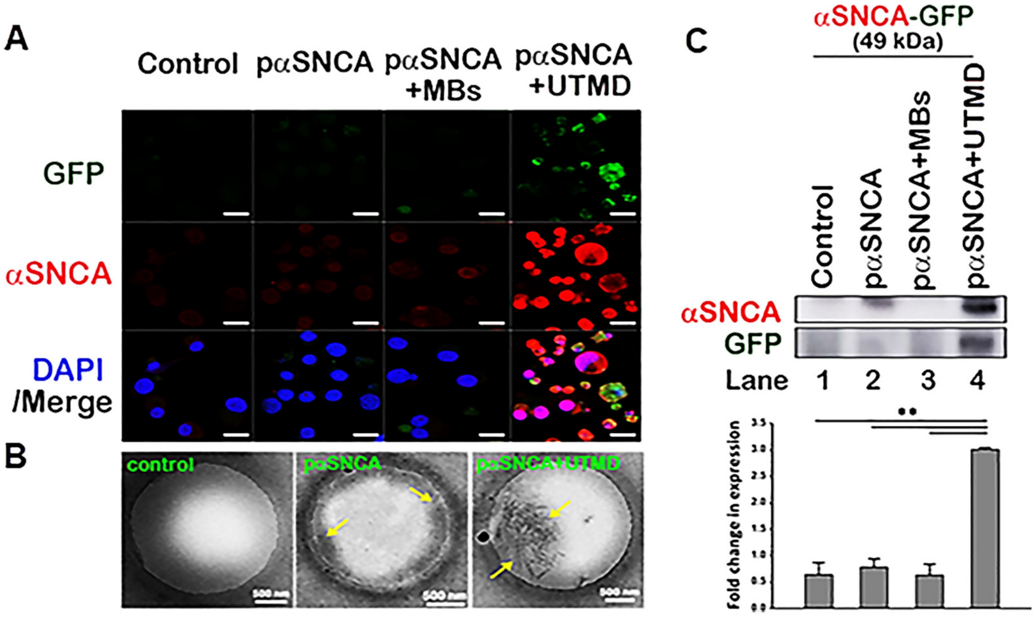

Figure S1. (**A**) Generation of αSNCA-GFP-expressing N2A. Combination of GFP fluorescence (green) and indirect immunofluorescence for αSNCA (red). Overlapped signals are yellow or orange. Scale bars = 10 μm. (**B**) CyroEM images of αSNCA fibril morphology. (**C**) Immunoblots detecting αSNCA and GFP expression levels for different experimental groups. There was significant difference according to the Mann-Whitney U test, $^{**}p < 0.01$. pαSNCA = plasmid α-synuclein; MBs = microbubbles; UTMD = ultrasound-targeted microbubble destruction.

Protein Aggregation-Induced Cytotoxicity

To study the physiological changes after gene transfection, we performed the ICC for caspase 3, MDA, BCL2 and BAX on N2A cells. The signaling that resulted when we introduced pDNA into cells was distributed evenly throughout the cytoplasm in the pαSNCA+UTMD group.

At the same time, we noted a weak signal in the control group, the pαSNCA-only group, and the pαSNCA+MBs group (Figure S2A). Caspase 3 is a hallmark of the late apoptotic marker, and we observed that the UTMD-induced αSNCA overexpression increased caspase 3 activity. We also observed that MDA was overexpressed, suggesting that the aggregates markedly induced oxidative stress. Meanwhile, both BCL2 (related to anti-apoptosis) and BAX (related to apoptosis) were present, and the data showed that BCL2 and BAX levels were higher in pαSNCA+UTMD cells than in the cells of the other conditions. Collectively, these results indicate that αSNCA induced oxidative stress and apoptosis in vitro after UTMD had established reproducible transfection in N2A cells.

We then evaluated whether UTMD could affect N2A differentiation. For this evaluation, we observed the expression levels of Tuj1 and GFAP, which are widely used biomarkers for neuronal cells and glial cells. We used dual-labeled ICC (green: Tuj1 for neurons, GFAP for glia; blue: DAPI for all cell nuclei) staining to colocalize GFP expression (green). Both the Tuj1-positive cells and GFAP-positive cells exhibited greater fluorescent intensities in the pαSNCA+UTMD group than in the control group, the pαSNCA group, and the pαSNCA+MBs group (Figure S2B). Morphologically, the Tuj1-positive cells colocalized with GFP expression in N2A cells and exhibited longer and more complex neurite outgrowths than was the case with the other cells. Similarly, the GFAP marker was used to identify the glial cells. Both the control group and the pαSNCA-only group exhibited only short protrusions; no genuine neurites were observed. In the pαSNCA+MBs group without US exposure, a temperature-mediated weak shock wave was identified as the leading cause, and we confirmed that the adhesion of MBs and pαSNCA to the cells was occasional but minimal for inducing a transient state of cell-membrane permeability. These results indicate that sonoporation-gene delivery promoted neural cell differentiation, inducing neurite outgrowth.

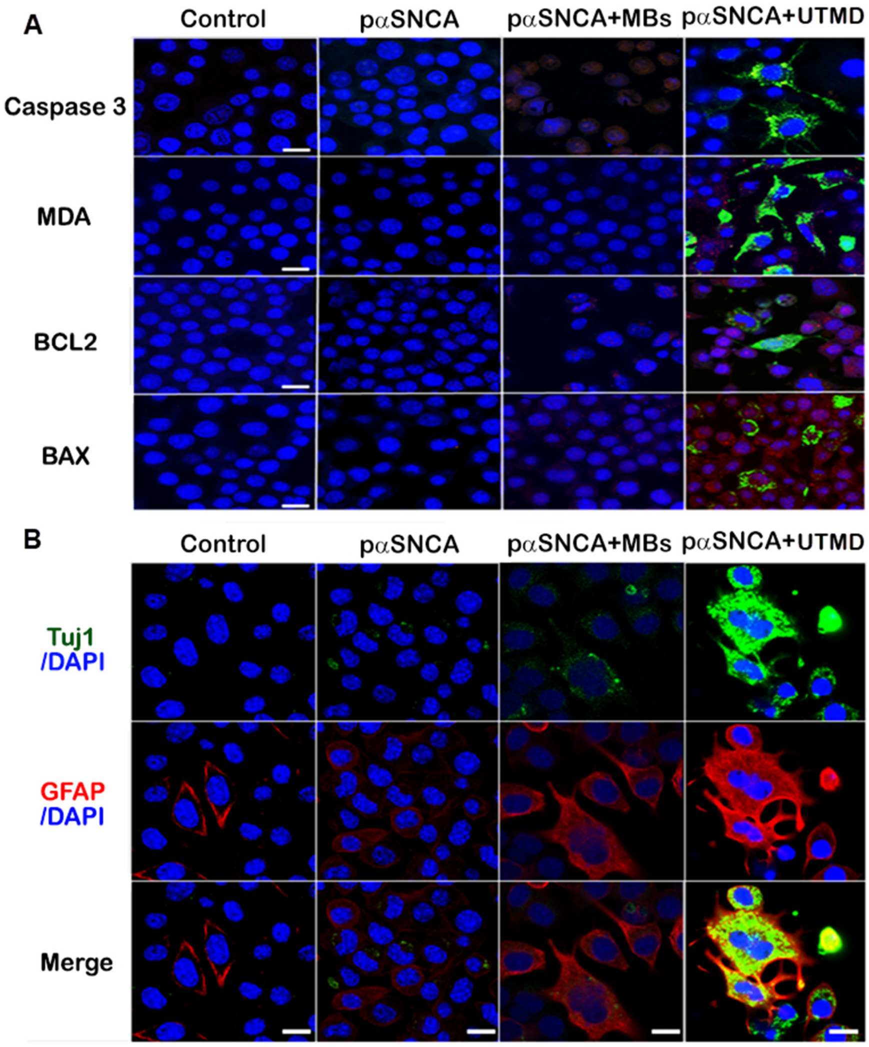

Figure S2 (**A**) Expression levels of Caspase 3, MDA, BCL2, and BAX in transfected N2A cells in the experimental groups. Caspase 3, MDA, BCL2 and BAX (red); GFP (green); DAPI (the nucleus, blue). (**B**) Effects of transfection efficiency in N2A cells. Fluorescence images showing the localization of Tuj1 immunoreactivity (green), GFP-expressing cells (green), GFAP immunoreactivity (red), and DAPI (blue). Statistically significant difference was done with the Mann-Whitney U test; $^{**}p < 0.01$. Scale bars = 10 μm.

In Vivo Protein Expression

Protein lysates were analyzed from brain tissues for immunoblotting analysis (Figure S3). The quantitation of αSNCA and GFP levels was analyzed via immunoblotting. We double confirmed significantly higher levels of GFP and αSNCA expression on days 2 and 7 with UTMD than in the controls (*$p < 0.05$ on both day 2 and day 7).

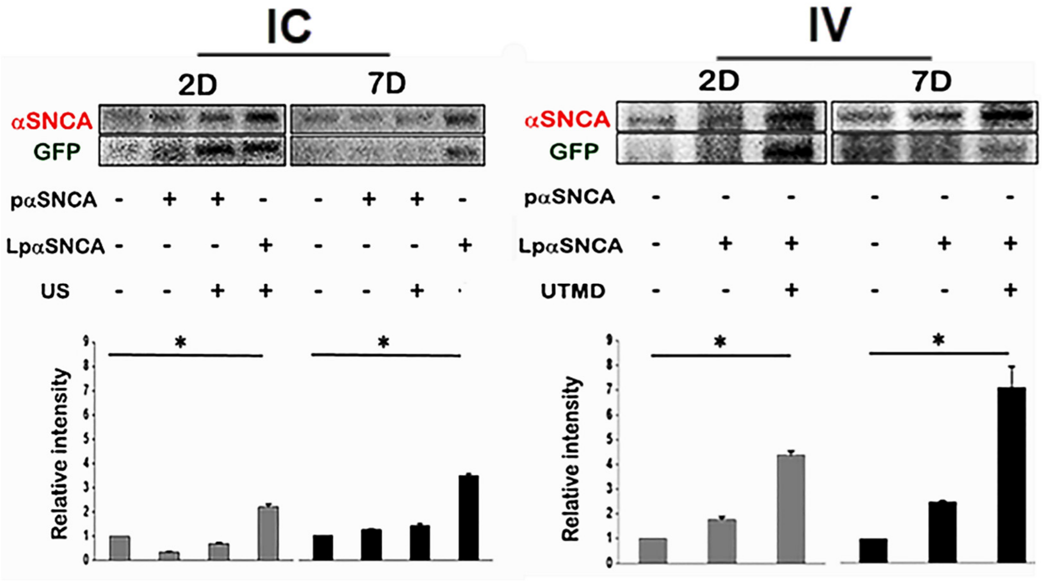

Figure S3. Representative protein expression following IC with naked pαSNCA, LpαSNCA or LpαSNCA +/− US expressed at day 2 and day 7, and IV with LpαDNA +/− UTMD expressed on day 2 and day 7. Relative intensity compared to the control. *$p < 0.05$ (Mann-Whitney U test). US = focused ultrasound; UTMD = focused ultrasound-targeted microbubble destruction; ROI = region of interest; IC = intracerebral; IV = intravenous; D = day. Arrows indicate aggregates. Scale bars = 10 µm.

Supplementary References

1. Luk, K.C., Song, C., O'Brien. P., et al. Exogenous alpha-synuclein fibrils seed the formation of Lewy body-like intracellular inclusions in cultured cells. *Proc. Natl. Acad. Sci. USA.* **2009**, *106*, 20051–20056, doi: 10.1073/pnas.0908005106.

2. Li, B., Ge, P., Murray, K.A., et al. Cryo-EM of full-length α-synuclein reveals fibril polymorphs with a common structural kernel. *Nat. Commun.* **2018**, 9, 3609, doi: 10.1038/s41467-018-05971-2.
